# The Charity Organisation Society

**Published:** 1921-09-10

**Authors:** 


*To the Editor of* The Hospital.

### The Charity Organisation Society.

*To the Editor of* The Hospital.

Sir,—I read with very real regret that the Charity Organisation Society is obliged, owing to lack of funds, to close one of its branch offices, and, to my considerable surprise, I now hear that it is the one working in Lewisham.

Whatever views one may hold as to Charity Organisation Society principles and the way in which they are applied, there can be no two opinions as to the soundness of their aims and the thoroughness of their work. It is only too easy in these days to obtain doles from charity or the State for any person in whom one is interested, but after experience of social workers throughout the length and breadth of London and in the provinces I have yet to find a Society which will deal systematically with a "case" from start to finish and from all its aspects, unless it be the Charity Organisation Society or a Society working on their lines, or a worker trained by them.

Acknowledging that the "vision" which enables the worker to interpret successfully their high and very broad principles is often lacking; that one feels sometimes that (possibly owing to the lack of necessary funds) they refuse help to applicants who might "derive permanent benefit" from it; that the Society stands for ideas which we are trying to-day (apparently in vain) to ignore or disprove, I think we must still regret as a real loss to the community the curtailing of the work of a Society which has inspired the selfless labours and held the devotion of a large number of thoughtful workers for the good of mankind, and which is still acknowledged to give a practical grounding unequalled by that of any other body to those who propose to try their hand at helping that complex being, their fellow-man.

That a large and, in part, well-to-do borough such as Lewisham and the neighbourhood should be unable or unwilling, if the want were more widely known, to raise the few hundreds a year needed to enable the Charity Organisation Society office to be kept open appears almost incredible.—Yours faithfully, D. D. D.

